# Impact of High-Dose Caffeine on the Preterm Ovine Cerebrum and Cerebellum

**DOI:** 10.3389/fphys.2019.00990

**Published:** 2019-08-02

**Authors:** Anzari Atik, Robert De Matteo, Meghan Boomgardt, Sandra Rees, Richard Harding, Jeanie Cheong, Shreya Rana, Kelly Crossley, Mary Tolcos

**Affiliations:** ^1^Department of Anatomy and Developmental Biology, Monash University, Clayton, VIC, Australia; ^2^The Ritchie Centre, Hudson Institute of Medical Research, Department of Obstetrics and Gynecology, Monash University, Clayton, VIC, Australia; ^3^Department of Anatomy and Neuroscience, The University of Melbourne, Melbourne, VIC, Australia; ^4^Department of Neonatal Services, Royal Women’s Hospital, Victorian Infant Brain Studies, Murdoch Children’s Research Institute, and Department of Obstetrics and Gynaecology, The University of Melbourne, Melbourne, VIC, Australia; ^5^School of Health and Biomedical Sciences, RMIT University, Bundoora, VIC, Australia

**Keywords:** apnea of prematurity, cerebral cortex, sheep, striatum, neurodevelopment

## Abstract

Caffeine is one of the few treatments available for infants with apnea of prematurity. As the recommended dosing regimen is not always sufficient to prevent apnea, higher doses may be prescribed. However, little is currently known about the impact of high-dose caffeine on the developing brain; thus, our aim was to investigate the consequences of a high-dose regimen on the immature ovine brain. High-dose caffeine (25 mg/kg caffeine base loading dose; 20 mg/kg daily maintenance dose; *n* = 9) or saline (*n* = 8) was administered to pregnant sheep from 105 to 118 days of gestation (DG; term = 147 days); this is broadly equivalent to 28–33 weeks of human gestation. At 119DG, the cerebral cortex, striatum, and cerebellum were assessed histologically and by immunohistochemistry. Compared with controls, caffeine-exposed fetuses showed (i) an increase in the density of Ctip2-positive layers V–VI projection neurons (*p* = 0.02), Tbr1-positive layers V–VI projection neurons (*p* < 0.0001), astrocytes (*p* = 0.03), and oligodendrocytes (*p* = 0.02) in the cerebral cortex, (ii) a decrease in the density of Cux1-positive layers II–IV projection neurons (*p* = 0.01) in the cerebral cortex, and (iii) a reduction in the area of Purkinje cell bodies in the cerebellum (*p* = 0.03). Comparing high-dose caffeine-exposed fetuses with controls, there was no difference (*p* > 0.05) in: (i) the volume of the cerebral cortex or striatum, (ii) the density of neurons (total and output projection neurons) in the striatum, (iii) dendritic spine density of layer V pyramidal cells, (iv) the density of cortical GABAergic interneurons, microglia, mature oligodendrocytes or proliferating cells, (v) total cerebellar area or dimensions of cerebellar layers, or (vi) the density of cerebellar white matter microglia, astrocytes, oligodendrocytes, or myelin. Daily exposure of the developing brain to high-dose caffeine affects some aspects of neuronal and glial development in the cerebral cortex and cerebellum in the short-term; the long-term structural and functional consequences of these alterations need to be investigated.

## Introduction

Caffeine therapy has become an integral part of management of apnea of prematurity in very preterm infants. Infants who do not respond to the standard recommended caffeine treatment (20 mg/kg caffeine citrate (loading) followed by 5–10 mg/kg daily) may require higher doses (Scanlon et al., 1992; Steer et al., 2003; Mohammed et al., 2015). However there is a paucity of evidence to show that doses above the “standard dose” are safe for the developing brain. We have previously reported that daily high-dose caffeine had no detectable effects on the cerebral white matter (WM) of the immature ovine brain (Atik et al., 2014). Here we report the effects of high-dose caffeine on the developing cerebral cortex, striatum, and cerebellum in a clinically appropriate animal model.

Previous studies have shown both beneficial (Juarez-Mendez et al., 2006; Connolly and Kingsbury, 2010) and detrimental effects (Fuller et al., 1982; Kang et al., 2002; Desfrere et al., 2007; Black et al., 2008) of caffeine on the developing brain, using a range of animal models. Thus the impact of caffeine on the developing brain at the cellular level remains contentious. Reported beneficial effects of high-dose caffeine exposure include increased total dendritic length and arborization of layer III pyramidal neurons of the prefrontal cortex of rats (Juarez-Mendez et al., 2006) and reduced seizure susceptibility to some chemo-convulsants in both juvenile and adult rats (Guillet and Dunham, 1995). Caffeine administered to rat pups significantly reduced brain injury caused by hypoxia-ischemia, as indicated by a reduction in neuronal necrosis and infarction (Bona et al., 1995). Caffeine has also been associated with adverse effects on the developing brain including a reduction in cell proliferation in the subventricular zone (SVZ) and dentate gyrus, a reduction in astrocytogenesis in the cerebral cortex (Desfrere et al., 2007), and increased apoptosis throughout the cerebral hemispheres (Kang et al., 2002; Black et al., 2008). In preterm infants, high-dose caffeine treatment during the first 24 h of life increases the incidence of cerebellar hemorrhage compared with infants treated with standard doses of caffeine, with no apparent injury to the cerebellar white or gray matter (McPherson et al., 2015).

Previous studies on the neurodevelopmental consequences of caffeine exposure have largely been conducted in rodents, a species in which key aspects of brain development differ from humans in their timing relative to birth. Furthermore, these studies have used different regimens of caffeine administration, including a variety of doses, routes and durations, making it difficult not only to extrapolate findings to preterm infants, but also to draw conclusions on the cellular effects of high-dose caffeine. Here we have used sheep, a long-gestation species in which the gestational timing of major events in brain development is similar to that in humans. Importantly neurodevelopmental events occurring in the sheep fetus are similar to those occurring in the very preterm human infant.

It is likely that a degree of cerebral hypoxia is present in preterm infants with apnea of prematurity; this is often difficult to quantify precisely, and the direct association between acute intermittent hypoxia and specific brain injury is speculative. Here, and in a previous study (Atik et al., 2014), we have sought to examine the effects of 2 weeks of high-dose caffeine exposure on the developing brain, without the potentially confounding effects of cerebral hypoxia. We have already demonstrated that such exposure does not affect the microstructure of the cerebral WM (Atik et al., 2014). In the present study we assessed aspects of the microstructure of the cerebral cortex, striatum and cerebellum as these brain regions are critical to cognition, memory and learning, and neuronal loss and/or gliosis in these regions could lead to long-term deficits in neurocognitive function (Pierson et al., 2007). Importantly, deficits in these key aspects of brain function have been reported in very preterm infants (Saigal and Doyle, 2008).

## Materials and Methods

### Ethics Statement

All experimental procedures were approved by the Monash University Animal Ethics Committee.

### Experimental Protocol

Date-mated pregnant ewes (Merino × Border Leicester, *n* = 14) underwent aseptic surgery at 99 days of gestation (DG; term is approximately 147DG) for implantation of catheters into a fetal femoral artery and vein, the amniotic sac and a maternal jugular vein as previously described (Atik et al., 2014). Five days after the surgery, a loading dose of caffeine base (25 mg/kg maternal weight, maternal i.v., Sigma-Aldrich, St. Louis, MO, United States) was administered to 9 of the ewes carrying either male or female fetuses, on 104DG (0.7 of term) followed by a daily maintenance dose (20 mg/kg maternal weight, maternal i.v.) from 105 to 118DG (0.7–0.8 of term; broadly equivalent to 28–33 weeks of human gestation) (Atik et al., 2014); control ewes received i.v. saline (*n* = 8, male and female fetuses). The caffeine dosing regimen was chosen in consultation with clinicians involved in the project and represents higher than normal doses which are similar to those currently being used in some neonatal intensive care units (Steer et al., 2003). It is necessary to administer caffeine to the ewe (based on maternal body weight) as our initial experiments revealed that caffeine administered directly to the fetus was rapidly lost to the ewe’s circulation via placental transport; intravenous infusion of caffeine (25 mg/kg followed by 20 mg/kg caffeine for 2 days) directly to the fetus via its femoral vein increased fetal plasma caffeine concentrations by < 0.1 mg/L. We administered caffeine base rather than caffeine citrate to minimize the volume required. Caffeine citrate contains anhydrous citric acid and 50% anhydrous caffeine base; thus the dose of caffeine base is approximately half that of caffeine citrate. Caffeine concentrations, body and organ weights, and physiological parameters were measured and have been reported previously (Atik et al., 2014). In brief, caffeine exposure did not alter fetal body weight, arterial pressure or oxygenation; the maximal fetal plasma caffeine concentration achieved (32 mg/L) was high relative to the range of concentrations measured in preterm infants treated with a standard dose. Both treatment groups received the same feed (lucerne chaff [export quality]; Southern Cross Feeds).

### Tissue Collection

At 119DG (0.8 of term), ewes and fetuses were euthanized using an overdose of sodium pentobarbitone (130 mg/kg i.v.) and the fetuses delivered via Caesarean section. Fetal brains were transcardially perfused *in situ* with isotonic saline and 4% paraformaldehyde in 0.1M phosphate buffer (pH 7.4). The entire forebrain was cut coronally into blocks 5 mm thick (8–10 blocks per animal) and the cerebellum was bisected at the midline of the vermis. Blocks of the entire right cerebral and right cerebellar hemispheres were then post-fixed in 4% paraformaldehyde (4 days, 4°C) and embedded in paraffin. The analyses described below have been applied to fetal brains obtained in a previous study (Atik et al., 2014) but are entirely different from the analyses previously reported.

### Histology and Immunohistochemistry

Serial sections (8 μm thick) were cut from each block of the cerebral and cerebellar hemispheres and 1 section per block stained with thionin or hematoxylin and eosin (cerebellum only) and qualitatively examined for hemorrhages and gross structural alterations. One section from equivalent sites from each of the four lobes of the right cerebral hemisphere (four sections per animal) and two sections of the cerebellum (separated by 80 μm) from each animal were immunostained to identify mature neurons (NeuN; cerebral hemispheres only), post-mitotic projection neurons in layers II–IV (cut-like homeobox gene, Cux1; cerebral hemispheres only), layers V–VI (Ctip2; cerebral hemispheres only), and layers V–VI [Tbr1; predominantly layer VI projection neurons (Hevner et al., 2001); cerebral hemispheres only], GABAergic interneurons (SST; cerebral hemispheres only), microglia (Iba-1), astrocytes (GFAP), oligodendrocytes within the entire lineage (Olig2), mature myelinating oligodendrocytes and myelinated axons (MBP), proliferating cells (Ki67; cerebral hemispheres only) and Purkinje cells (Calbindin; cerebellum only) (Table 1). Immunostaining was performed using the avidin-biotin complex elite kit (Vector Laboratories, Burlingame, CA, United States) as previously described (Atik et al., 2014). For each antibody, sections from control and caffeine-treated animals were stained simultaneously to reduce variability. There was no staining when the primary antibodies were omitted.

**TABLE 1:** Immunohistochemistry: primary and secondary antibodies.

**1° antibody and dilution**	**Localizes**	**Supplier**	**2° antibody and dilution**
^*^Mouse anti-NeuN 1:500	Mature neurons	MAB377; Millipore, Billerica, MA, United States	Biotinylated goat anti-mouse IgG; 1:200
^*#^Rat anti-Ctip2 1:500	Projection neurons in cortical layers V–VI	ab28448, Abcam, Cambridge, MA, United States	Biotinylated anti-rat IgG; 1:200
^†^^#^Rabbit anti-Cux1 1:200	Projection neurons in cortical layers II–IV	Orb156497, Biorbyt, Cambridge, United Kingdom	Biotinylated anti-rat IgG; 1:200
^#^Rabbit anti-Tbr1 1:100	Projection neurons in cortical layers V–VI	AB10554, Millipore, Billerica, MA, United States	Biotinylated goat anti-rabbit IgG; 1:200
^Rabbit anti-SST 1:500	GABAergic interneurons	A0566, DAKO, Carpinteria, CA, United States	Biotinylated anti-rabbit IgG; 1:200
^Mouse anti-Calbindin 1:500	Purkinje cells in cerebellum	SWANT	Biotinylated goat anti-mouse IgG; 1:200
^*#^Rabbit anti-Ki67 1:200	Proliferating cells in late G1, S, G2 and M phases	RM9106, Thermo Scientific, Waltham, MA, United States	Biotinylated anti-rabbit IgG; 1:200
^*#^Rabbit anti-Iba-1 1:1500 (cerebrum); 1:1000 (cerebellum)	Microglia	019-19741, WAKO Pure Chemical Industries, Osaka, Japan	Biotinylated anti-rabbit IgG; 1:200
^*#^Rabbit anti-GFAP 1:1000	Astrocytes	ZO2334, DAKO, Carpinteria, CA, United States	Biotinylated anti-rabbit IgG; 1:200
^*^Rabbit anti-Olig2 1:500	Oligodendrocytes	AB9610, Millipore, Billerica, MA, United States	Biotinylated anti-rabbit IgG; 1:200
^*^Rat anti-MBP 1:100	Mature myelinating oligodendrocytes	MAB395, Millipore, Billerica, MA, United States	Biotinylated anti-rat IgG; 1:200

Pre-treated with ^*^0.01 M citrate buffer (pH6) and microwaving; ^†^Tris EDTA (pH9), heated in water bath; ^0.02% proteinase K (30 min, 37°C); ^#^counterstained with 0.01% thionin.

### Golgi-Cox Staining

For each fetus, one block from the left cerebral hemisphere (approximately section 1160 according to the Michigan State University sheep brain atlas^1^) was processed for Golgi-cox impregnation (FD Rapid Golgi Stain Kit; FD Neurotechnologies, Inc., Columbia, MD, United States). Blocks were serially sectioned (100 μm thick) in the coronal plane with a cryostat (Leica CM3050, Leica Microsystems, Pty Ltd., Australia). Sections were mounted onto gelatin-coated slides, processed for Golgi visualization using materials supplied in the kit, dehydrated in graded alcohols and coverslipped.

### Histological and Immunohistochemical Analysis

**FIGURE 1:**
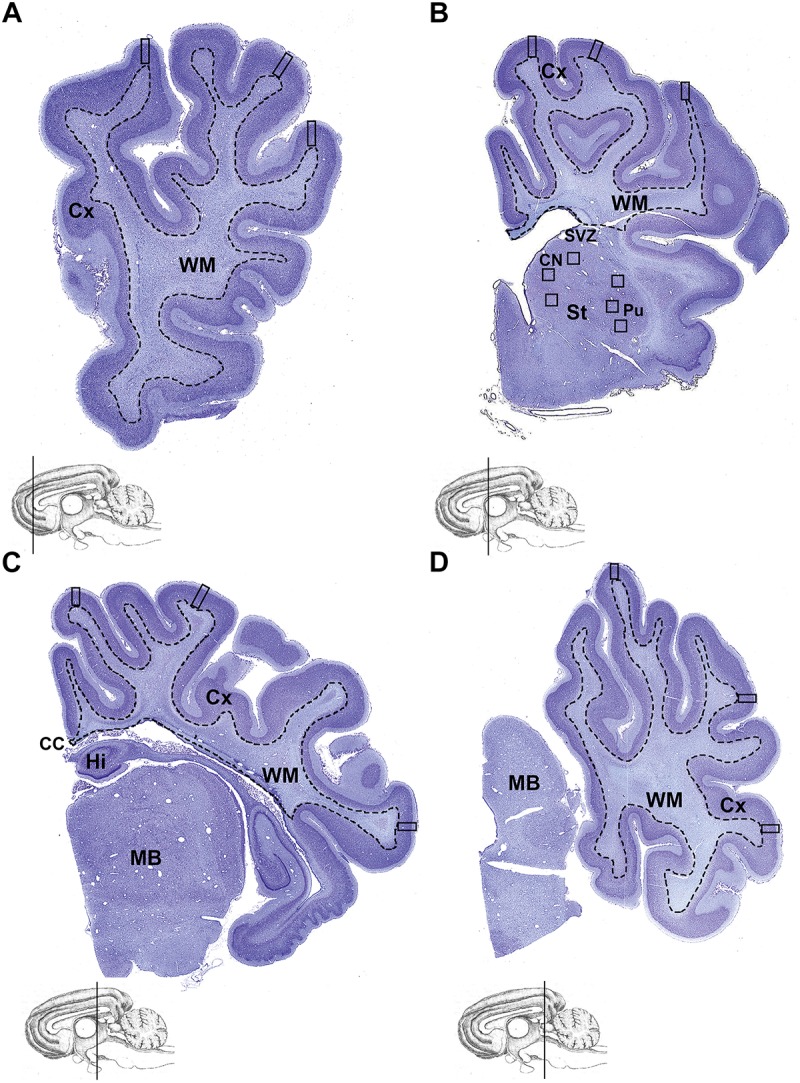
Brain regions analyzed. Coronal, thionin-stained, hemi-sections of the cerebral hemisphere at the level of the **(A)** frontal, **(B)** parietal, **(C)** temporal, and **(D)** occipital lobe. Cerebral cortex (Cx; regions outside the dashed line) was examined for all histological and immunohistochemical analysis. Measurements were made in the cerebral cortex (squares indicate fields of view; 4 bins analyzed per square for NeuN; 3 bins analyzed per square for Ctip2; whole square without bins analyzed for SST). A total of 6 fields of view was analyzed from the striatum, with 3 fields of view selected from the caudate nucleus and 3 fields from the putamen **(B)**. Cx, cortex; CC, corpus callosum; Hi, hippocampus; St, striatum; SVZ, subventricular zone; CN, caudate nucleus; Pu, putamen; MB, midbrain.

Analyses were performed on coded slides (observer blinded to group) from the right cerebral hemisphere and cerebellum (at the level of the vermis) using an image analysis system (ImageScope, Aperio Technologies, San Diego, CA, United States). Immunohistochemical analyses were performed on one section from each of the frontal, parietal, temporal, and occipital lobes (Ki67, parietal and temporal only) from each fetus; the same region within the gray matter and striatum was assessed in each animal (Figure 1). For the cerebellum, all analyses were performed on two sections per animal, and assessments made in early (lobule I or X) and late (lobule VII or VIII) developing lobules (Altman, 1969), and the deep WM, in randomly selected fields with the exception of total cross-sectional cerebellar areal measurements (see below). Data from early and late developing lobules and the deep WM were combined and averaged, except for the assessment of cerebellar layers. For each immunostain, the mean cell density was calculated for each fetus and for each region or bin, and a mean of means for control and caffeine-treated groups determined.

#### Volumetric Analysis of the Cerebral Cortex and Striatum

The cross-sectional areas of the cerebral cortex and striatum were separately measured in every 625^th^ thionin-stained section (1 section per 5 mm block; n = 8 sections per animal) using a digitizer interfaced to the image analysis software (Tolcos et al., 2011). The total volume of the cerebral cortex and striatum were estimated according to the Cavalieri principle using the formula V = ΣAPt, where V is the total volume, ΣA is the sum of the areas measured, P is the inverse of the sampling fraction and t is the section thickness (Gundersen et al., 1999).

#### Morphological Assessment of the Cerebellum

Cerebellar sections were scanned for signs of hemorrhage or overt damage. The cross-sectional area of the vermis was measured in two sections of the cerebellum (separated by 80 μm). Tracing of the total cerebellar area was performed at x4 objective on Aperio scanned slides. Automated area (mm^2^) of the total cross-section of the cerebellum, the IGL and the WM was calculated by ImageScan Scope software. Area of the IGL and WM were expressed as a ratio of the total cerebellum area. The external granule layer (EGL) represents the transient progenitor site from which granule cells arise and thus differences in EGL width following high-dose caffeine administration would be indicative of developmental disturbances. In order to assess differences in the width of the EGL between control and caffeine cohorts, 40 sites per section were measured from the early developing (I or X) and late developing (VII or VIII) lobules (80 sites/cerebellum). The width of the ML offers a surrogate measurement for assessing connectivity, as this layer is largely comprised of the Purkinje cell dendritic outgrowth as well as the parallel fibers of the granule cells. In order to assess the differences in the width of the ML following high-dose caffeine administration, 40 sites per cerebellar section were measured from the early developing (I or X) and late developing (VII or VIII) lobules (80 sites/cerebellum). Purkinje cell somal areas were measured in 40 calbindin-positive Purkinje cells (with a clearly defined cell body and nucleus) randomly selected from the early developing (I or X) and late developing (VII or VIII) lobules (80 cells/cerebellum). The mean Purkinje cell somal area was calculated for each fetus (late and early lobules combined), and a mean of means for control and caffeine-treated groups determined.

#### Analysis of Neurons, Glia, and Proliferating Cells in the Cerebral Cortex and Striatum

For each fetus, NeuN-immunoreactive (IR) neurons were counted in the striatum (3 fields in the caudate and 3 in putamen, total of 6 fields/section/fetus; field size, 0.40 mm^2^) (Figure 1); assessment of NeuN-IR neuronal density in the cerebral cortex has previously been reported (Atik et al., 2014). Ctip2-IR neurons were counted in the cerebral cortex (1 field/gyrus, total of 3 fields/section/fetus, field size, 0.56 mm^2^); each field was further divided into 3 bins (bin 1: cortical layer I; bin 2: layers II, III, and IV; bin 3: layers V and VI). Ctip2-IR neurons were also counted in the striatum (6 fields/section/fetus; field size, 0.40 mm^2^). Cux1- and Tbr1-IR neurons were counted in layers II–IV (Cux1) or V–VI (Tbr1) (1 field/sulcus, total of 3 fields/section/fetus; field size, 0.75 mm^2^). SST-IR interneurons were counted in 0.40 mm wide columns that spanned all 6 layers of the cerebral cortex (3 fields/gyrus; total of 12 fields/section/fetus; field size, 0.56 mm^2^) and in the striatum (6 fields/section/fetus; field size, 0.40 mm^2^).

Iba-1-IR microglia, GFAP-IR astrocytes, Olig2-IR oligodendrocytes, MBP-IR mature myelinating oligodendrocytes and Ki67-IR proliferating cells were counted in random fields of view throughout the cerebral cortex (12 fields/section/fetus; field size, 0.140 mm^2^); Ki67-IR cells were also counted in the SVZ (9 fields/section/fetus; field size, 0.140 mm^2^).

#### Analysis of the Dendritic Spine Density of Pyramidal Cells in the Cerebral Cortex

Dendritic spines were counted along the apical dendrite (50 μm segment and ∼5 μm from the soma) of layer V pyramidal cells (5 cells/one section/fetus) in region-matched, Golgi-stained sections from control (n = 6) and caffeine-treated (n = 7) fetuses (x100 magnification; oil immersion). The mean number of spines/10 μm segment of dendrite was calculated for each fetus and group means were obtained by averaging the mean values of each fetus. Pyramidal neurons were selected for analysis when they met the following criteria: (i) triangular-shaped soma with an apical dendrite perpendicular to the pial surface; (ii) complete impregnation of the cell with Golgi-stain that permitted visualization of the entire dendritic arbor; and (iii) processes were not obscured by other neurons, glia, or the vasculature. Basal dendrites were excluded from analysis due to the large degree of overlap with other dendrites.

### Analysis of Glia and Myelination in the Cerebellum

In the cerebellum, the areal density of Iba-1-IR microglia and Olig2-IR oligodendrocytes were assessed in the WM [deep WM and lobular WM combined; 8 fields/section/fetus; field size, 0.36 mm^2^ (deep WM) and 0.12 mm^2^ (lobular WM)]. The intensity of MBP-IR and GFAP-IR in the cerebellar WM was determined using a validated optical density (OD) analysis as described previously (Atik et al., 2014). From each section, OD was assessed in 3 fields from equivalent regions of the deep WM, 2 fields from equivalent regions of the WM from early developing (I or X) lobules, and 2 fields from equivalent regions of the WM from late developing (VII or VIII) lobules (total of 7 fields from each section). A correction was applied to each of these images by subtracting the OD measurement from a region of background staining. The mean OD was then calculated within each region, for each animal, and a mean of means determined for control and caffeine-treated animals. Imaging and analysis of each of the immunostains was performed in a single day using identical parameters to maintain consistency and eliminate error.

#### Qualitative Assessment of GFAP-IR in the Cerebellum

The morphology of GFAP-IR Bergmann glia was assessed qualitatively for evidence of fiber disorganization (tangled or irregularly orientated fibers), and disrupted fiber integrity (damaged/degenerating fibers and disconnected fibers: i.e., those that did not extend the full length from the IGL to the upper EGL) as previously described (McDougall et al., 2017; Tolcos et al., 2018). The density of GFAP-IR astrocytes in the IGL and ML was also qualitatively assessed.

### Statistical Analysis

All data were analyzed using SPSS software (Version 20, SPSS, Inc., Chicago, IL, United States). Differences between treatment groups were analyzed by the Student’s *t*-test for parametric data; if data failed a variance test (*F*-test), a Mann–Whitney *U*-test (non-parametric data) was used. Data are presented as mean of means ± SEM with *p* < 0.05 considered significant.

## Results

### Cerebral Cortex and Striatum

#### Volumetric Analysis

Fetal exposure to high-dose caffeine did not affect the volumes of cortical gray matter (control: 6.74 ± 0.25 cm^3^; caffeine: 7.16 ± 0.56 cm^3^; *p* > 0.05) or striatum (control: 0.37 ± 0.05 cm^3^; caffeine: 0.41 ± 0.04 cm^3^; *p* > 0.05).

#### Neurons and Pyramidal Cell Dendritic Spine Density

**FIGURE 2:**
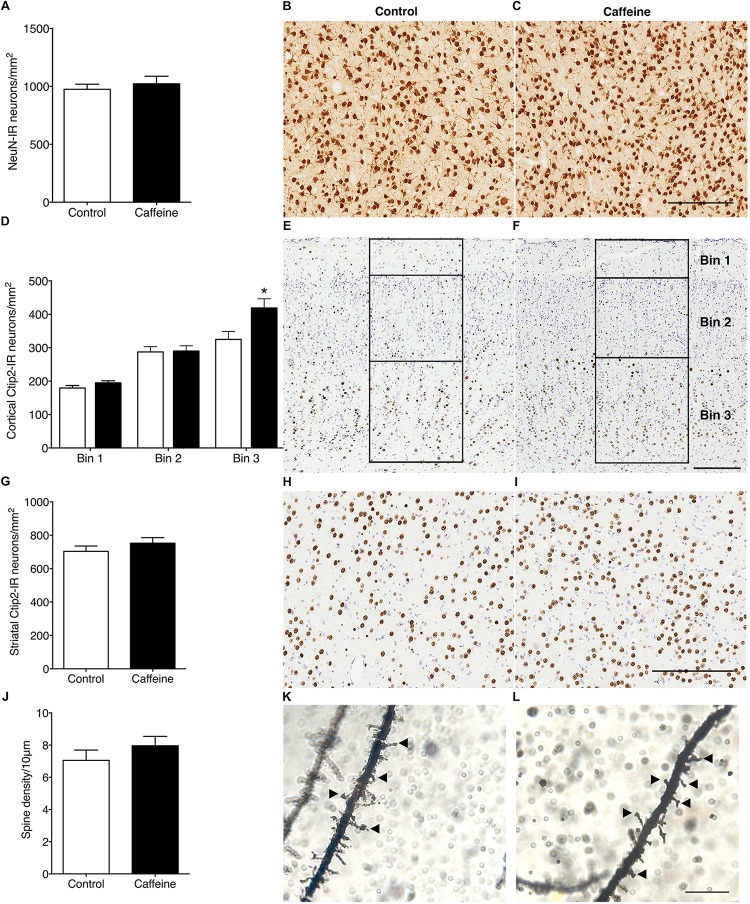
NeuN-IR neurons, Ctip2-IR projection neurons and layer V pyramidal neuron dendritic spine density in control and caffeine-treated fetuses. NeuN-IR neuronal density in the striatum **(A)** was not different between control and caffeine-treated fetuses, illustrated by comparing images from the striatum in control **(B)** and caffeine-treated **(C)** fetuses at 119DG. There was a significant increase in Ctip2-IR projection neurons in bin 3 (layers V and VI) in the cerebral cortex **(D)**, in the caffeine group compared to controls, with no significant differences in all other bins. This is illustrated by comparing images of Ctip2-immunoreactivity in control **(E)** and caffeine-treated **(F)** fetuses. Bins were divided according to cortical layers (bin 1: cortical layer I; bin 2: layers II, III, and IV; bin 3: layers V and VI) as shown in **(E,F)**. There was no significant difference between control and caffeine-treated fetuses in Ctip2-IR neuronal density within the striatum **(G)**, illustrated by comparing images from the striatum of control **(H)** and caffeine-treated **(I)** fetuses. There was no significant difference between control and caffeine-treated fetuses in dendritic spine density per 10 μm of apical dendrite **(J)**. This is illustrated by comparing high power images of Golgi-cox stained sections from the cerebral cortex in control **(K)** and caffeine-treated **(L)** fetuses at 119DG. Arrowheads show dendritic spines on apical dendrites. Scale bar **(B,C,E,F,H,I)** = 200 μm; **(K,L)** = 10 μm. ^*^p < 0.05.

The density of NeuN-IR neurons in the striatum was not different (*p* > 0.05) in control and caffeine-treated fetuses (Figures 2A–C). In the cerebral cortex, caffeine treatment led to a significantly greater density of Ctip2-IR neurons in bin 3 (layers V and VI) (*p* = 0.02; Figures 2D–F), with a tendency for an increase when all cortical bins were combined (*p* = 0.07; data not shown); in the striatum there was no difference (*p* > 0.05) between groups (Figures 2G–I). There was a significant decrease in the density of Cux1-IR neurons (control: 123.3 ± 8.5 cells/mm^2^; caffeine: 86.5 ± 9.7 cells/mm^2^; *p* = 0.01), and a significant increase in the density of Tbr1-IR neurons (control: 40.83 ± 1.1 cells/mm^2^; caffeine: 65.5 ± 2.0 cells/mm^2^; *p* < 0.0001) in the cerebral cortex of caffeine treated fetuses compared with controls.

**FIGURE 3:**
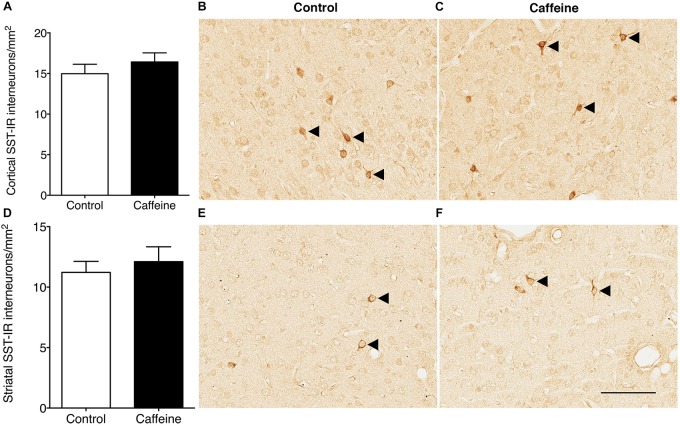
SST-IR interneurons in the cerebral cortex and striatum of control and caffeine treated fetuses. SST-IR interneuron density was not affected by high-dose caffeine compared to controls in the cerebral cortex **(A)** or striatum **(D)**. This is illustrated by comparing images from the cerebral cortex **(B,C)** and striatum **(E,F)** in control **(B,E)** and caffeine-treated **(C,F)** fetuses. Arrowheads show SST-positive cells. Scale bar **(C–F)**: 100 μm.

There was no significant difference (*p* > 0.05) between groups in the linear density of dendritic spines on layer V pyramidal cells (Figures 2J–L). There was no qualitative difference between groups in the length or shape of the spines. The density of SST-IR interneurons was not different (*p* > 0.05) between groups in the cerebral cortex (Figures 3A–C) or striatum (Figures 3D–F).

#### Glia and Cell Proliferation

**FIGURE 4:**
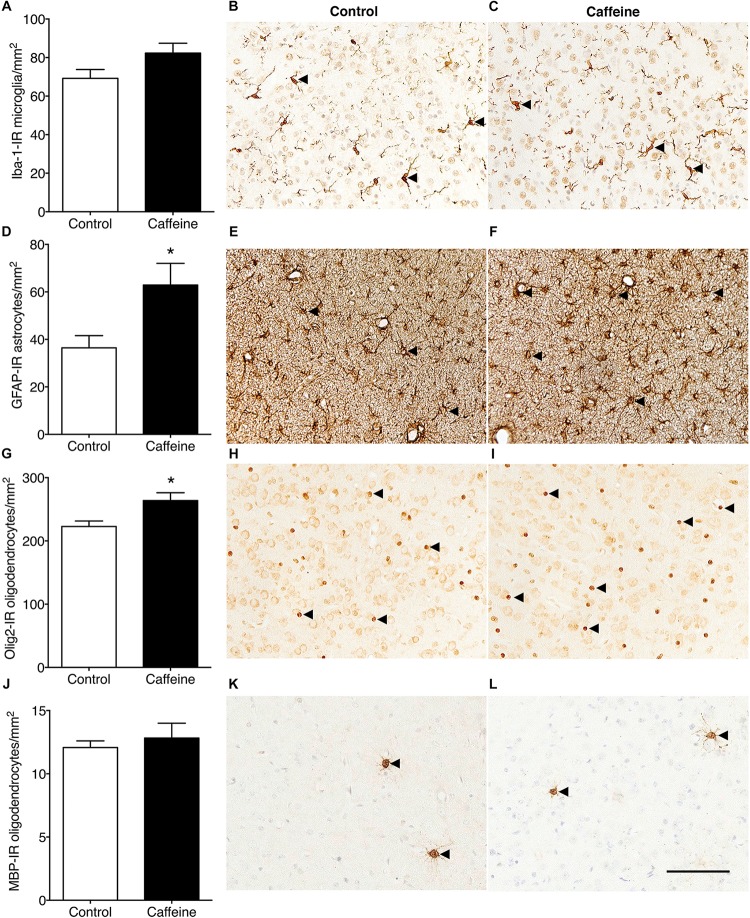
Iba-1-IR microglia, GFAP-IR astrocytes and Olig2-IR and MBP-IR oligodendrocytes in the cerebral cortex of control and caffeine-treated fetuses. The density of Iba-1-IR microglia (resting and activated combined) was not different in caffeine-treated and control fetuses **(A)**, illustrated by comparing images from the cerebral cortex in control **(B)** and caffeine-treated **(C)** fetuses. There was a significant increase in the density of GFAP-IR astrocytes in caffeine-treated fetuses compared to controls **(D)**, evident by comparing images from the cerebral cortex in control **(E)** and caffeine-treated **(F)** fetuses. There was a significant increase in the density of Olig2-IR oligodendrocytes in the cerebral cortex in caffeine-treated fetuses compared to controls **(G)**. This is shown by comparing images from the cerebral cortex in control **(H)** and caffeine-treated **(I)** fetuses. The density of MBP-IR mature myelinating oligodendrocytes was not different in the cerebral cortex of caffeine-treated and control fetuses **(J)**, illustrated by comparing images from the cerebral cortex of control **(K)** and caffeine-treated **(L)** fetuses. In **(B,C)**, arrowheads show ramified microglia; in **(E,F)** arrowheads show astrocytes; in **(H,I)** arrowheads show Olig2-IR oligodendrocytes and in **(K,L)** arrowheads show MBP-IR mature oligodendrocytes. Scale bar = 100 μm. ^*^p < 0.05.

**FIGURE 5:**
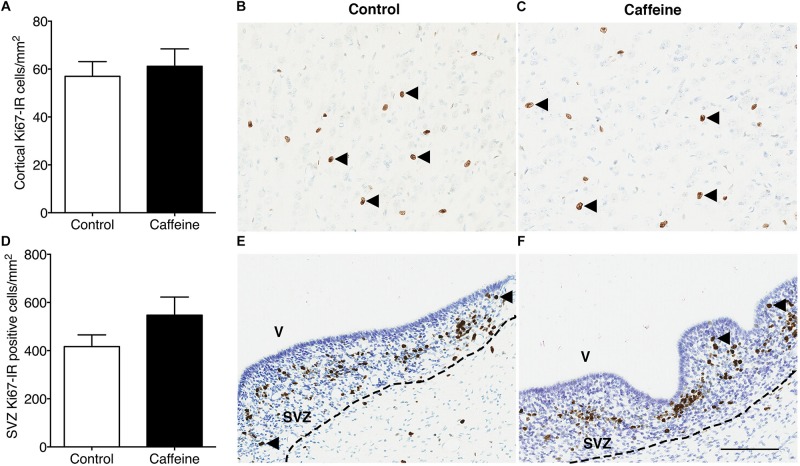
Ki67-IR cells in the cerebral cortex and subventricular zone of control and caffeine-treated fetuses. The density of Ki67-IR cells in cerebral cortex **(A)** or subventricular zone **(D)** did not differ between control and caffeine-treated fetuses. This is illustrated by comparing images from the cerebral cortex **(B,C)** and subventricular zone **(E,F)** of control **(B,E)** and caffeine-treated **(C,F)** fetuses. Arrowheads show Ki67-IR cells. V, ventricle; SVZ, subventricular zone. Scale bar **(B,C,E,F)** = 100 μm.

The density of Iba-1-IR microglia in the cerebral cortex did not differ (*p* > 0.05) between treatment groups (Figures 4A–C). Caffeine exposure led to an increase in the density of cortical GFAP-IR astrocytes (*p* = 0.03; Figures 4D–F) and an increase in the density of cortical Olig2-IR oligodendrocytes (*p* = 0.02; Figures 4G–I). There was no difference in the areal density of MBP-IR mature myelinating oligodendrocytes (Figures 4J–L) or Ki67-IR proliferating cells in cerebral cortex (Figures 5A–C), or in the density of Ki67-IR cells in the SVZ (Figures 5D–F) in control compared to caffeine-treated fetuses (*p* > 0.05).

### Cerebellum

No hemorrhages or overt structural damage were observed. There was no significant difference in cerebellum weight (control: 3.0 ± 0.1 g; caffeine: 3.0 ± 0.1 g) or the ratio of cerebellum weight to brain weight (control: 0.08 ± 0.003; caffeine: 0.07 ± 0.002).

#### Morphological Analysis

In caffeine-treated fetuses compared to controls there was no difference (*p* > 0.05) in the total cross-sectional area of the cerebellum (control: 119.6 ± 5.1 mm^2^; caffeine: 127.0 ± 6.9 mm^2^; *p* > 0.05), the ML (control: 44.4 ± 1.8 mm^2^; caffeine: 48.1 ± 3.4 mm^2^; *p* > 0.05), the IGL (control: 47.6 ± 1.9 mm^2^; caffeine: 49.4 ± 3.5 mm^2^; *p* > 0.05), or WM (control: 27.7 ± 5.1 mm^2^; caffeine: 29.5 ± 1.4 mm^2^; *p* > 0.05), nor in the width of the EGL (control: 26.1 ± 1.2 μm; caffeine: 28.0 ± 0.4 μm; *p* > 0.05). There was however a reduction (*p* < 0.05) in the mean Purkinje cell somal area (control: 491.0 ± 11.1 μm^2^; caffeine: 439.9 ± 17.4 μm^2^; *p* = 0.03).

#### Glia and Myelination

In the WM, there was no difference (*p* > 0.05) in the areal density of Iba-1-IR microglia (control: 131.4 ± 22.6 cells/mm^2^; caffeine: 126.1 ± 14.3 cells/mm^2^; *p* > 0.05), Olig2-IR oligodendrocytes (control: 2449 ± 54.5 cells/mm^2^; caffeine: 2464 ± 154.2 cells/mm^2^; *p* > 0.05), nor in the OD of MBP-IR (control: 0.22 ± 0.006; caffeine: 0.22 ± 0.007; *p* > 0.05) or GFAP-IR (control: 0.14 ± 0.004; caffeine: 0.13 ± 0.004; *p* > 0.05) between groups. Qualitative analysis of GFAP-IR in the ML and IGL showed no difference between groups. There were also no observable differences in the morphology of the Bergmann glia fibers between groups: i.e., no evidence of fiber disorganization or disrupted fiber integrity.

## Discussion

This is the first study to assess the effects of high-dose caffeine on the developing cerebral cortex in a long-gestation, clinically relevant animal model. Key findings were that in the developing cortex, high-dose caffeine led to an increase in the density of Ctip2-IR projection neurons in layers V–VI, Tbr1-IR projection neurons in layers V–V1, and a decrease in Cux1-IR projection neurons in the upper cortical layers (II–IV). There was also an increase in the density of cortical GFAP-IR astrocytes and Olig2-IR oligodendrocytes but no change in dendritic spine density of layer V pyramidal neurons, nor in the density of interneurons, microglia, mature oligodendrocytes, or proliferating cells. Caffeine exposure had no effect on any of the neuronal and glial parameters measured in the striatum and cerebellum apart from a small but significant reduction in mean Purkinje cell somal area.

### Effects of High-Dose Caffeine on Pyramidal Neurons in the Cerebral Cortex

We previously reported that high-dose caffeine treatment does not affect neuronal density within the cerebral cortex (Atik et al., 2014); here we report that the volume of the cerebral cortex is also unaffected, making it unlikely that there are major changes in overall neuronal numbers. However to determine whether specific neuronal populations had been differentially affected, we identified excitatory pyramidal projection neurons of layers V–V1 using antibodies to Trb1 and Ctip2, a zinc-finger transcription factor; Ctip2 is more strongly expressed in sub-cortically projecting neurons than in inter- and intra-hemispherically projecting neurons (Arlotta et al., 2005). In addition we identified projection neurons in the upper layers (layers II–IV) with antibodies to Cux1, a transcription factor which regulates several genes involved in cellular proliferation and differentiation (Nepveu, 2001). We found that caffeine treatment resulted in an increase in the density of Ctip2-IR and Tbr1-IR neurons in the deeper cortical layers (early born projection neurons) and a decrease in Cux1-IR neurons in the upper layers (late born projection neurons). As this occurred in the absence of an overall increase in neuronal density, it suggests an upregulation in the expression of these proteins in previously low expressing cells.

As Ctip2 is a transcription factor critical for axonal outgrowth and extension as well as for axonal pathfinding by cortical projection neurons (Arlotta et al., 2005; Leyva-Diaz and Lopez-Bendito, 2013), an increase in Ctip2-immunoreactivity could lead to aberrant growth of subcortically projecting axons. This could occur at the expense of cortically projecting axonal growth as has been shown in rodents, when there is an imbalance of transcription factors (Alcamo et al., 2008; Britanova et al., 2008). Although we have not exhaustively examined developmental gene expression patterns it is clear that caffeine treatment results in alterations to neurochemical signatures which could affect appropriate axonal growth and target finding (Arlotta et al., 2005; Leyva-Diaz and Lopez-Bendito, 2013). The functional significance of these alterations is likely complex and calls for further investigation but underlines the need for examination at the cellular level when determining whether a specific regime has affected neuronal function. Although we found no change in the overall density of SST-IR (inhibitory) interneurons in the cerebral cortex of caffeine-treated fetuses, it should be noted that the SST-expressing interneurons are only one subpopulation of GABAergic interneurons, comprising approximately 23% of the entire GABAergic interneuron population (Gonchar et al., 2007); thus other populations may be differentially affected by caffeine.

### High-Dose Caffeine and Synaptic Connectivity in the Cerebral Cortex

To determine the effects of high-dose caffeine on synaptic connectivity, we assessed the linear density of spines on the apical dendrites of layer V pyramidal neurons. We found no effect on spine density but recognize that other parameters of dendritic morphology such as the overall extent of the dendritic arbor need to be assessed. A study of the effects of caffeine exposure on dendritic morphology in newborn rats similarly showed no differences in spine density; however an increase in dendritic length was observed (Juarez-Mendez et al., 2006).

### Effects of High-Dose Caffeine on Neuroglia in the Cerebral Cortex

We previously reported that high-dose caffeine does not result in an increase in the areal density of microglia, astroglia, or oligodendroglia in the developing ovine cerebral WM (Atik et al., 2014). Here we report that, in the cerebral cortex, the same regimen resulted in astrogliosis in the absence of microgliosis; however the mechanism underlying this cortical gliosis is uncertain. Astrogliosis is commonly associated with neural damage induced by an insult such as cerebral hypoxia (Brand and Bignami, 1969). Cerebral hypoxia may have occurred in our animals although we have previously shown that fetal arterial oxygen saturation and arterial pressure were unaffected throughout the treatment period; however, cerebral blood flow was not measured (Atik et al., 2014). Caffeine is known to decrease cerebral blood flow (Tracy et al., 2010) and acts as a neuronal stimulant (Nehlig et al., 1992), possibly increasing neuronal metabolic demand; both factors operating in concert could cause cerebral hypoxia, affecting neuronal survival. However, we saw no evidence of neuronal apoptosis and neuronal densities were not reduced in our model (Atik et al., 2014), although it is possible that cell death may have occurred at an earlier time point. In preterm baboons administered caffeine to stimulate breathing, cortical gliosis was also present in the absence of overt cell death (Loeliger et al., 2006). The authors (Loeliger et al., 2006) argued that caffeine might increase the vulnerability of cortical gray matter to intermittent hypoperfusion while preserving the cerebral WM. Further studies are required to determine whether cortical astrogliosis persists in the long-term in our model. Such persistence could have implications for neural function as astrocytes can either be beneficial or detrimental to neural tissue (Sofroniew and Vinters, 2010). Our findings are not in accord with those of Desfrere et al. (2007) who found transient and dose-dependent inhibition of astrocytogenesis in the developing mouse cortex following postnatal caffeine exposure; the different findings are possibly due to differences in species, dosing regimen, or timing of exposure.

In addition to astrogliosis, we have shown that high-dose caffeine increased the areal density of Olig2-IR oligodendrocytes in the cerebral cortex. There was no evidence of increased cell proliferation in the cerebral cortex or SVZ to account for the increased density; however, caffeine might have influenced the proliferation of oligodendrocytes at an earlier time point than the one examined in this study. The fate of this increased pool of Olig2-IR cells is uncertain, as it was not associated with an increase in the density of myelinating oligodendrocytes. A proportion of cells in the oligodendrocyte lineage in the cerebral cortex become perineuronal satellite cells (Szuchet et al., 2011) and are thought to provide support to neurons, possibly via regulation of the microenvironment (Dewar et al., 2003). Perineuronal oligodendrocytes also maintain a reservoir of untranslated transcripts encoding major myelin proteins (Szuchet et al., 2011). It is possible that the density of this population of oligodendrocytes is increased as a result of high-caffeine exposure but we were not able to investigate this.

### Effects of High Dose Caffeine on the Cerebellum

The degree of caffeine exposure that we used had no effect on the cerebellar parameters measured here apart from a small (∼10%) but significant decrease in the area of the Purkinje cell body. Such a decrease could ultimately have an effect on dendritic growth but the lack of a reduction in the area of the ML (of which the Purkinje cell dendritic arbor is a major component) does not support the likelihood of such an outcome. We have previously shown that the most rapid expansion of the Purkinje cell dendritic tree in fetal sheep occurs between 100 and 120DG (Rees and Harding, 1988), coinciding with the time course of the present study. If caffeine exposure were to affect dendritic growth we would have expected to identify it during this period of rapid expansion.

### Are There Long-Term Developmental Outcomes Following High-Dose Caffeine Administration?

Overall, caffeine exposure causes some relatively subtle but specific changes in the developing cerebral cortex while having a minimal effect in the cerebellum and no effect in the striatum; the reason for this differential effect is not clear and does not appear to be related to different developmental stages of the regions (Rees et al., 1997). Whether or not the subtle changes that we report in neuronal and glial cell development persist postnatally, or manifest into long-term functional deficits is unknown. In humans, clinical trials assessing the immediate and longer-term implications of high-dose caffeine therapy for apnea of prematurity are now emerging. Magnetic resonance imaging and neurobehavioral testing undertaken at term equivalent age, and at 2 years of age, showed that very preterm infants who received high-dose (80 mg/kg caffeine citrate; loading dose) compared with standard dose (20 mg/kg loading dose) caffeine, had a higher incidence of cerebellar hemorrhage with subsequent alterations in motor performance (McPherson et al., 2015).

## Conclusion

Using an animal model in which the brain is at a stage of development resembling that of the very preterm infant, we have shown that daily administration of high-dose caffeine for 2 weeks affects aspects of neuronal and glial cell development in the cerebral cortex but has minimal effects on the cerebellum and no effect on the striatum. Although the long-term structural and functional consequences of our findings are not yet known, our data, coupled with recent adverse findings in preterm infants (McPherson et al., 2015), highlight the need to consider both the benefits of high-dose caffeine [e.g., improved extubation from mechanical ventilation (Steer et al., 2004; Mohammed et al., 2015)], and potentially adverse effects. It is therefore important that investigators continue to study the neurodevelopmental impact of high-dose caffeine in clinically relevant, long-gestation animal models.

## Data Availability

The datasets generated for this study are available on request to the corresponding author.

## Author Contributions

RH, JC, SaR, RDM, and MT designed the experiments. AA, RDM, ShR, MB, KC, and MT collected the data. AA, ShR, and MB analyzed the data. AA, RH, JC, SaR, RDM, MB, KC, and MT interpreted the data. AA, RH, RDM, SaR, and MT wrote and revised the manuscript. All authors approved the final version of the manuscript.

## Conflict of Interest Statement

The authors declare that the research was conducted in the absence of any commercial or financial relationships that could be construed as a potential conflict of interest.

## Footnotes

1 https://www.msu.edu/∼brains/brains/sheep/index.html

## Abbreviations

CC: corpus callosum
CN: caudate nucleus
Ctip2: COUP-TF interacting protein 2
Cux1: cut-like homeobox 1
Cx: cortex
DG: days of gestation
EGL: external granule layer
GFAP: glial fibrillary acidic protein
Hi: hippocampus
Iba-1: ionized binding adaptor molecule-1
IGL: internal granule layer
IR: immunoreactive
MB: midbrain
MBP: myelin basic protein
ML: molecular layer
NeuN: neuronal nuclei
OD: optical density
Olig2: oligodendrocyte transcription factor 2
Pu: putamen
St: striatum
SST: somatostatin
SVZ: subventricular zone
Tbr1: T-box brain 1
WM: white matter.
